# Evaluation of Pulmonary Functional Complications After COVID-19 Infection in Children: A Retrospective and Prospective, Observational Study

**DOI:** 10.3390/jcm15124624

**Published:** 2026-06-14

**Authors:** Aleksandra Bareła, Agnieszka Blomberg, Aleksandra Filipczak, Jerzy Arendarczyk, Oskar Sylwestrzak, Krzysztof Zeman, Marcin Tkaczyk

**Affiliations:** 1 Department of Pediatrics, Immunology and Nephrology, Institute of the Polish Mother’s Health Center in Lodz, 93-338 Lodz, Poland; 2 Department of Pediatrics, Nephrology and Immunology, Medical University of Lodz, 93-338 Lodz, Poland; 3 Department of Obstetrics and Gynecology, Institute of the Polish Mother’s Health Center in Lodz, 93-338 Lodz, Poland

**Keywords:** COVID-19, SARS-CoV-2, children, spirometry, pulmonary function, post-COVID complications

## Abstract

**Background:** More than 750 million cases of COVID-19 have been reported worldwide. The respiratory system, particularly the lungs, is one of the main targets of SARS-CoV-2 infection. Although persistent pulmonary function abnormalities have been described in adults, evidence in pediatric populations remains limited and inconsistent. Children usually experience a milder course of COVID-19; however, the long-term impact of SARS-CoV-2 infection on respiratory function in this group is still unclear. Current studies report conflicting findings regarding persistent spirometric abnormalities and their relationship with disease severity and time since infection. Therefore, further research is needed to better characterize post-infectious respiratory sequelae in children and adolescents. The aim of this study was to evaluate spirometric abnormalities in a pediatric cohort following COVID-19 infection. **Methods:** This retrospective and prospective observational study included 109 children and adolescents aged 6–18 years with a history of asymptomatic, mildly symptomatic, or symptomatic COVID-19 infection. Spirometry was performed following recovery from infection, and pulmonary function parameters were analyzed according to clinical course and time since infection. **Results:** Spirometry was conducted at a mean of 4.3 ± 2.8 months after infection. Abnormalities in pulmonary function were identified in 23.85% of the study population, with reduced FVC being the predominant spirometric abnormality. No statistically significant association was observed between the severity of COVID-19 and spirometric impairments (*p* > 0.5). Abnormal spirometry findings were observed across all post-infection time intervals examined; however, no statistically significant differences were identified between the groups. **Conclusions:** Spirometric abnormalities, predominantly reduced FVC, were observed in a substantial proportion of pediatric patients following SARS-CoV-2 infection. Although no clinical predictors were identified, the absence of pre-infection measurements and a control group limits interpretation. Longitudinal studies are required to clarify the clinical relevance and persistence of these pulmonary function changes.

## 1. Introduction

Since the beginning of the COVID-19 pandemic, more than 750 million SARS-CoV-2 infections and nearly 7.1 million deaths have been reported worldwide at the time of writing. Initially, children and adolescents under 18 years of age accounted for approximately 5% of confirmed cases, with this proportion increasing to 10–23% during subsequent waves of the pandemic [1]. Increasing evidence suggests that COVID-19 may lead to persistent post-infectious complications in a substantial proportion of affected individuals [2]. Therefore, the assessment of long-term consequences of SARS-CoV-2 infection in the pediatric population remains an important clinical issue.

The most frequently reported symptoms of COVID-19 include cough, dyspnea, fever, fatigue, headache, myalgia, gastrointestinal symptoms, and disturbances of taste and smell [3]. In children, the disease course is generally asymptomatic or mild to moderate [4]. Nevertheless, a small proportion of pediatric patients develop Paediatric Inflammatory Multisystem Syndrome Temporally Associated with SARS-CoV-2 (PIMS-TS), typically occurring 2–6 weeks after infection. At the peak of the pandemic, PIMS-TS was diagnosed in approximately 13 per 10,000 pediatric patients with SARS-CoV-2 infection [1]. The syndrome is characterized by persistent fever, elevated inflammatory markers, and evidence of single- or multi-organ involvement [5].

Current evidence from adult populations indicates that the lungs are among the organs most frequently affected by SARS-CoV-2 infection. COVID-19 may cause alveolar epithelial and capillary injury, subsequently leading to fibrosis and hyalinization [6,7]. As a result, persistent respiratory dysfunction lasting for months or even years after infection has been reported in adults [8]. Although these observations provide biologically plausible mechanisms for post-infectious pulmonary abnormalities, their direct applicability to pediatric populations remains uncertain, as children differ from adults in terms of disease severity, immune response, and baseline respiratory risk profiles.

By contrast, evidence on pulmonary sequelae of COVID-19 in children remains limited and inconclusive. In a large Italian cohort of 433 patients aged 0–18 years who underwent spirometry after SARS-CoV-2 infection, no significant impairment in pulmonary function was observed, and spirometric parameters did not differ according to the time elapsed since infection [9]. However, this study predominantly included children with asymptomatic or mild disease, while patients with moderate-to-severe respiratory involvement were not represented. This limits the generalizability of the available data and highlights an important clinical gap regarding pulmonary function across different levels of COVID-19 severity and at varying time intervals after infection. Therefore, the present study aimed to evaluate respiratory function in children and adolescents during the first months following SARS-CoV-2 infection, with particular attention to spirometric parameters in relation to disease severity, time since infection, and selected anthropometric characteristics, including body mass index (BMI).

## 2. Materials and Methods

The study population consisted of 109 children and adolescents aged 6–18 years with a previous history of SARS-CoV-2 infection.

The sample size was determined by the number of eligible patients recruited during the study period. Given the observational design of the study, no formal a priori sample size calculation was performed. As an illustrative reference, a post hoc estimate based on a chi-square comparison across three groups, assuming α = 0.05, 80% power, and a medium effect size (Cohen’s w = 0.30), indicates that approximately 106 participants would be required. The final sample comprised 109 children; however, the study may have been underpowered to detect small effects, and the results of multivariable analyses should be interpreted with appropriate caution.

The study had a retrospective and prospective observational design. Participants were enrolled at a mean of approximately 4 months after SARS-CoV-2 infection. Eligibility criteria included age between 6 and 18 years and confirmed SARS-CoV-2 infection, defined by a positive antigen or RT-PCR test during the acute phase of illness, or by the presence of anti-SARS-CoV-2 IgG antibodies in unvaccinated individuals. Serological testing was performed exclusively in symptomatic patients for whom the date of symptom onset could be reliably established, whereas asymptomatic patients were diagnosed solely on the basis of positive antigen or RT-PCR test results. In addition, the study included data from patients previously hospitalized who had documented SARS-CoV-2 infection and underwent pulmonary function testing.

The date of infection was defined according to the diagnostic modality. For participants diagnosed by RT-PCR or antigen testing, the date of the first positive test was considered the date of infection. In serology-confirmed cases, the reported date of symptom onset was used as a proxy for the date of infection. Time from infection to spirometry was calculated as the interval between the defined date of infection and the date of pulmonary function testing.

Patients were recruited at the Department of Pediatrics, Immunology and Nephrology of the Polish Mother’s Memorial Hospital Research Institute (ICZMP) between January 2021 and November 2022. The study protocol was approved by the institutional Bioethics Committee (approval no. 45/2021, issued on 18 May 2021) and covered both the retrospective and prospective parts of the study. Written informed consent was obtained from the parents or legal guardians of all participants prior to enrollment.

The severity of COVID-19 infection was classified according to World Health Organization (WHO) criteria. Participants were categorized as asymptomatic (*n* = 10), mildly symptomatic (*n* = 60), or symptomatic (*n* = 39). The severity of SARS-CoV-2 infection was assessed retrospectively based on detailed medical history obtained from patients and their parents during clinical evaluation, as well as available medical records from the acute phase of infection, including hospital discharge summaries and outpatient documentation when available. Mildly symptomatic infection was defined by the presence of symptoms such as fever, cough, or fatigue without evidence of pneumonia or hypoxemia, and without the need for hospitalization. Symptomatic infection included patients presenting with clinical features of pneumonia, dyspnea, respiratory distress, or reduced oxygen saturation [10,11].

Anthropometric measurements, including body mass index (BMI), were assessed according to age- and sex-specific WHO percentile charts. Nutritional status was classified as follows: underweight (<5th percentile), normal weight (5th–85th percentile), overweight (>85th percentile), and obesity (≥95th percentile) [12].

Pulmonary function testing was performed using a MES Lungtest 1000 spirometer (MES Sp. z o.o., Cracow, Poland), in accordance with the American Thoracic Society/European Respiratory Society (ATS/ERS) 2019 recommendations and Global Lung Function Initiative (GLI) reference standards [13,14].

Each participant performed a minimum of three spirometry maneuvers, with at least two technically acceptable and repeatable measurements required for analysis. The assessed spirometric parameters included forced vital capacity (FVC), forced expiratory volume in one second (FEV1), and the FEV1/FVC ratio (pseudo-Tiffeneau index). Examinations were considered technically valid when they fulfilled ATS/ERS quality criteria, including the absence of coughing, air leaks, or premature termination of expiration, as well as the achievement of reproducible flow-volume and volume-time curves [13,14]. No participant received bronchodilator medication prior to spirometric assessment. Bronchodilator reversibility testing was not performed, as the primary aim of the study was to evaluate baseline pulmonary function following SARS-CoV-2 infection rather than to investigate reversible airway obstruction. Furthermore, restricting the assessment to pre-bronchodilator spirometry minimized test duration and reduced procedural burden for the pediatric participants.

Spirometric results were interpreted by comparing measured values with reference standards adjusted for age, sex, height, and ethnicity. Percentile-based interpretation was applied to account for physiological developmental differences within the pediatric population. According to the ATS/ERS 2019 and Global Lung Function Initiative (GLI) recommendations, the lower limit of normal (LLN) was defined as the 5th percentile, while the upper limit of normal (ULN) corresponded to the 95th percentile [13,14]. Percentile values were generated automatically by the spirometry software based on GLI-derived z-scores. In accordance with GLI recommendations, values below the LLN corresponded to a z-score of less than −1.645 and were considered abnormal.

Medical history data regarding chronic respiratory conditions, including asthma and airway obstruction, were also collected and included in the analysis. To reduce the potential confounding effect of pre-existing pulmonary dysfunction on spirometric outcomes, patients with chronic respiratory diseases other than bronchial asthma were excluded from the study. Information regarding a prior diagnosis of bronchial asthma was collected and incorporated into the analysis due to its clinical relevance and prevalence within pediatric populations.

Binary indicator variables were created to identify spirometric parameters below the LLN. The following values were considered abnormal:

- FEV1 below the 5th percentile;
- FVC below the 5th percentile;
- FEV1/FVC ratio below the 5th percentile.

An overall spirometry result was classified as abnormal if at least one of the three principal spirometric parameters (FEV1, FVC, or FEV1/FVC) was below the LLN.

Statistical analyses were performed using nonparametric methods and regression models appropriate for binary outcome variables.

Continuous variables were summarized using descriptive statistics. Associations between quantitative variables were evaluated using Spearman’s rank correlation coefficient, a nonparametric measure of monotonic association that does not require the assumption of normal distribution [15].

Comparisons of the frequency of abnormal spirometry findings between categorical groups were conducted using Pearson’s chi-square test. When expected cell frequencies were below 5, Fisher’s exact test was applied [16].

Differences in spirometric percentile distributions among more than two independent groups were assessed using the Kruskal–Wallis test, a nonparametric alternative to one-way analysis of variance (ANOVA) [17].

Multivariate logistic regression analysis was performed to identify factors independently associated with abnormal spirometry results. Odds ratios (ORs) with corresponding 95% confidence intervals (CIs) were calculated for the analyzed predictors [18]. The goodness-of-fit of the logistic regression model was assessed using the Hosmer–Lemeshow test. Model discrimination was evaluated using the area under the receiver operating characteristic (ROC) curve. Multicollinearity among predictors was assessed using variance inflation factors (VIFs), with values below 5 considered acceptable.

A *p*-value < 0.05 was considered statistically significant. All statistical analyses were conducted using Python 3.12 with the pandas, numpy, scipy.stats, and statsmodels libraries.

## 3. Results

The study population consisted of 109 children and adolescents, including 58 boys and 51 girls, with a mean age of 11.9 years (range: 6–18 years). Pulmonary function assessment was performed at a mean of 4.3 months after SARS-CoV-2 infection (range: 1–14 months).

According to the WHO classification, 10 patients (9%) experienced an asymptomatic infection, 60 (55%) had a mildly symptomatic course, and 39 (36%) developed symptomatic COVID-19. The most commonly reported acute symptoms included fever, cough, dyspnea, pneumonia, gastrointestinal symptoms, pharyngitis and tonsillitis, disturbances of taste and smell, and fatigue.

Information regarding post-COVID complications and persistent symptoms was obtained during clinical interviews. The most frequently reported complaints included abdominal pain, skin lesions, chronic cough, reduced exercise tolerance, fatigue, dyspnea, recurrent respiratory tract infections, chest pain, persistent olfactory and taste disturbances, impaired concentration and memory, and musculoskeletal pain. As the assessment of post-COVID symptoms was descriptive in nature, no formal analysis of their association with spirometric abnormalities was performed.

The prevalence of abnormal spirometric parameters in the study group (*n* = 109) was as follows:

- FEV1 below the 5th percentile: 4/109 patients (3.7%);
- FVC below the 5th percentile: 17/109 patients (15.6%);
- FEV1/FVC ratio below the 5th percentile: 9/109 patients (8.3%).

Overall, abnormal spirometry findings were identified in 26 of 109 participants (23.85%).

Analysis of the pattern of abnormalities demonstrated that:

- Isolated reduction in FVC was present in 14 patients;
- Isolated reduction in FEV1/FVC was observed in 8 patients, including one patient with a history of bronchial asthma that remained clinically stable at the time of examination;
- Simultaneous reduction in FEV1 and FVC occurred in 3 patients;
- Simultaneous reduction in FEV1 and FEV1/FVC was identified in 1 patient.

The remaining 83 participants presented spirometric parameters within normal reference ranges for all three basic indices [Table 1].

The most frequently observed abnormality was a reduction in FVC, occurring both as an isolated finding and in combination with other abnormal spirometric parameters.

No statistically significant correlations were observed between age and spirometric parameters (*p* > 0.05).

Spirometric outcomes were subsequently compared according to the clinical severity of SARS-CoV-2 infection. No statistically significant differences were observed in the distribution of spirometric percentiles among asymptomatic, mildly symptomatic, and symptomatic patients (Kruskal–Wallis test, *p* > 0.5). Similarly, the prevalence of abnormal spirometry findings did not differ significantly between the analyzed groups (*p* = 0.28).

The relationship between time elapsed since SARS-CoV-2 infection and pulmonary function parameters was also evaluated. For this analysis, participants were divided into three groups according to the interval between infection and spirometry assessment:

- ≤3 months: 47 patients, including 10 with abnormal spirometry results;
- 4–6 months: 42 patients, including 13 with abnormal spirometry results;
- >6 months: 20 patients, including 3 with abnormal spirometry results.

**Figure 1:**
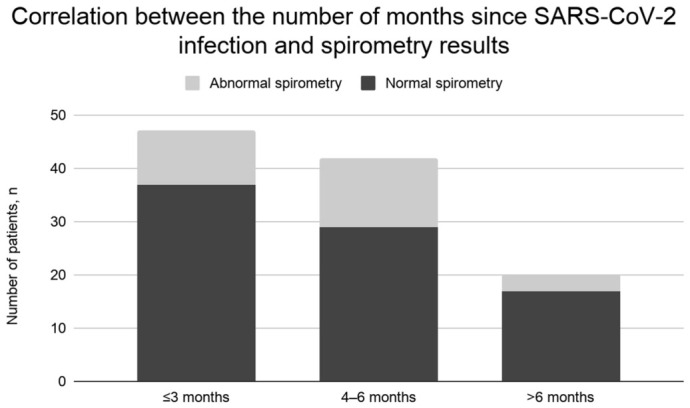
Frequency of normal and abnormal spirometry findings according to time since SARS-CoV-2 infection.

**Figure 2:**
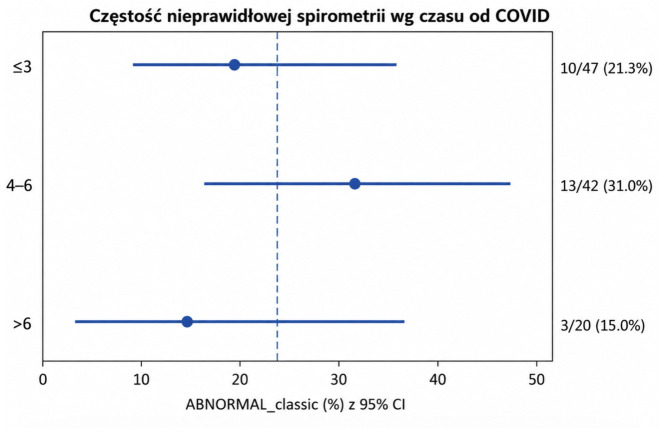
Frequency of abnormal spirometry results by time since COVID-19 infection.

Although the highest proportion of abnormal findings was observed within the first 6 months after infection, no statistically significant association was found between time since SARS-CoV-2 infection and spirometric abnormalities (*p* > 0.05) [Figure 1 and Figure 2].

**Figure 3:**
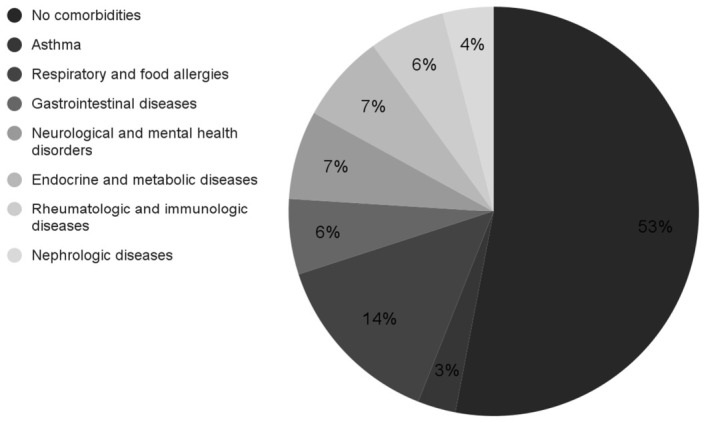
Frequency of comorbidities in study group.

Comorbidities were identified in 51 of 109 participants (46.8%), with at least one chronic condition reported in each of these patients. Bronchial asthma was present in 3 patients (2.8%). Other coexisting conditions included inhalant and food allergies in 15 patients (13.8%), gastroenterological disorders in 7 patients (6.4%), neurological and psychiatric disorders in 8 patients (7.3%), endocrine and metabolic diseases in 8 patients (7.3%), rheumatological and immunological disorders, including immunodeficiencies, in 7 patients (6.4%), and nephrological diseases in 5 patients (4.6%) [Figure 3].

**Figure 4:**
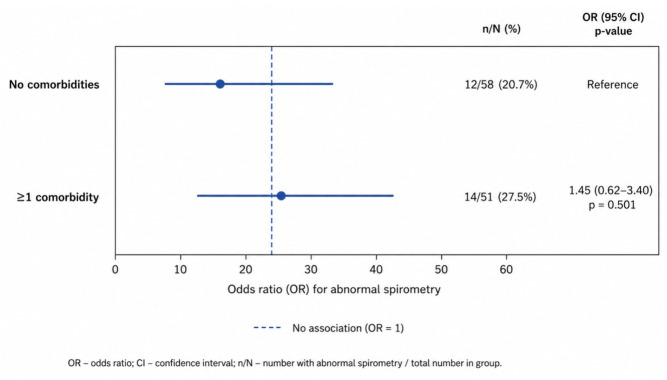
Plot of the relationship between the co-occurrence of chronic diseases and abnormal spirometric test results.

No statistically significant association was observed between the presence of comorbidities and the occurrence of abnormal spirometry results (Fisher’s exact test: *p* = 0.50; OR = 1.45) [Figure 4].

Body mass index (BMI) was assessed in all study participants. Underweight was identified in 5 patients (4.6%), while 84 participants (77.1%) had BMI values within the normal range (5th–85th percentile). Overweight (85th–95th percentile) was observed in 14 patients (12.8%), and obesity (≥95th percentile) in 6 patients (5.5%).

No statistically significant correlations were found between BMI percentile and individual spirometric parameters, including FEV1, FVC, and FEV1/FVC ratio.

In the multivariable logistic regression model using standardized BMI percentiles, BMI percentile remained independently associated with the risk of abnormal spirometry. A 10-percentile-point increase in BMI percentile was associated with lower odds of abnormal spirometry (OR = 0.80; 95% CI: 0.68–0.94; *p* = 0.007), after adjustment for age, time since SARS-CoV-2 infection, and COVID-19 clinical course. Age, time since infection, and COVID-19 clinical course were not significantly associated with abnormal spirometry.

**Figure 5:**
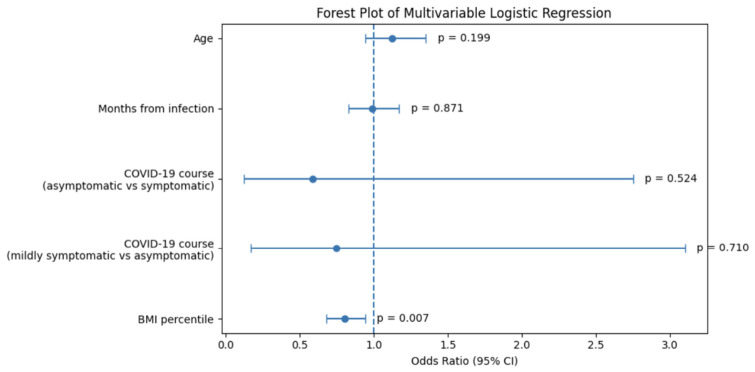
Forest Plot presenting odds ratio (OR) for age, time from infection, infection course, and BMI percentile.

The revised logistic regression model using BMI percentile showed acceptable calibration according to the Hosmer–Lemeshow test (χ^2^ = 5.19; df = 8; *p* = 0.738). The area under the ROC curve was 0.68, indicating modest discriminatory ability, and McFadden’s pseudo-R^2^ was 0.074. No relevant multicollinearity was detected, as all variance inflation factors for predictors were below 5 [Table 2, Figure 5].

## 4. Discussion

Since the SARS-CoV-2 pandemic, numerous studies have investigated the impact of COVID-19 on the health of both adult and pediatric populations, including its potential effects on the respiratory system. However, currently available data regarding pulmonary function abnormalities in children after SARS-CoV-2 infection remain inconsistent. Studies conducted by Di Chiara et al. and Bottino et al. in pediatric cohorts did not demonstrate significant impairment in pulmonary function following COVID-19 infection. Moreover, spirometric parameters remained within age-adjusted reference ranges regardless of disease severity [19,20].

In contrast, abnormal spirometry findings were identified in 23.85% of patients in our cohort. Reduced FEV1/FVC ratios were observed in 9 patients (8.3%), while FEV1 values below the 5th percentile were found in 4 patients (3.7%). The most frequent abnormality was reduced FVC (<5th percentile)—identified in 17 patients (15.6%).

Abnormal spirometry results were observed across all post-infection time intervals, with numerically higher proportions in participants evaluated ≤3 months and 4–6 months after SARS-CoV-2 infection (21.28% and 30.95%, respectively). However, these differences did not reach statistical significance, and no definitive temporal relationship can be inferred from the present data. Similar findings were reported by Sharanya et al., who observed pulmonary function abnormalities in children assessed within 1–6 months after SARS-CoV-2 infection [21]. In that cohort, abnormal spirometry was identified in approximately one-third of participants.

Potential mechanisms underlying post-COVID pulmonary dysfunction have been described by Varga et al. and Bridges et al. [22,23]. SARS-CoV-2 demonstrates a particular tropism for type II alveolar epithelial cells and ciliated airway epithelial cells. Viral-induced endothelial injury, disruption of the alveolar-capillary barrier, microvascular thrombosis, microangiopathy, and persistent inflammatory and cytokine-mediated processes may contribute to impaired respiratory function. These mechanisms may be associated with reduced lung volumes and lower FVC values on spirometry [22,23]. However, it should be emphasized that an isolated reduction in FVC suggests a possible restrictive pattern but does not confirm true pulmonary restriction, which requires verification by plethysmography and measurement of total lung capacity (TLC). Prolonged inflammatory processes within the respiratory epithelium may also promote tissue remodeling and fibrosis, which could contribute to the persistence of spirometric abnormalities for several months after infection [24]. Therefore, our findings support the need for long-term respiratory follow-up in pediatric patients after COVID-19, and additional imaging or more advanced functional testing may be considered in selected cases.

Among the children included in our study, three had a pre-existing diagnosis of bronchial asthma, and one of them demonstrated deterioration in pulmonary function following SARS-CoV-2 infection. Similar observations were reported by Chou et al., who described worsening spirometric parameters and poorer asthma control among patients with confirmed COVID-19 despite the overall improvement in asthma control reported in the general population during the pandemic [25]. Although this improvement has been attributed to increased hygiene measures, reduced exposure to inhaled allergens, and decreased contact with respiratory pathogens [25], SARS-CoV-2 infection itself may still adversely affect airway function in susceptible individuals. Airway epithelial injury and persistent inflammation have been proposed as potential mechanisms contributing to airway hyperresponsiveness and persistent respiratory symptoms following COVID-19 [26].

In the present study, no significant associations were identified between BMI and individual spirometric parameters, including FEV1, FVC, and the FEV1/FVC ratio. Nevertheless, multivariable logistic regression analysis demonstrated an association between higher BMI percentile and lower odds of abnormal spirometry after adjustment for age, time since SARS-CoV-2 infection, and COVID-19 severity. This observation should be interpreted with considerable caution. Given the limited number of overweight and obese participants, the exploratory nature of the analysis, and the potential influence of unmeasured confounding factors, the clinical significance of this finding remains uncertain. Furthermore, the observed association does not support any inference regarding a causal or protective effect of higher BMI on pulmonary function. Additional studies involving larger and more diverse pediatric cohorts are required to determine whether this association is reproducible and to clarify its potential clinical relevance.

Several limitations should be considered when interpreting the present findings. First, pulmonary function was assessed exclusively by spirometry. Although spirometry is a well-established, safe, reproducible, and widely available method for evaluating respiratory function, it provides only a limited characterization of pulmonary physiology. While it is useful for the early detection of ventilatory abnormalities and may serve as a screening tool, it does not allow for comprehensive assessment of the underlying pathophysiological mechanisms. In addition, the predominance of reduced FVC in the present cohort, together with evidence suggesting alveolar-capillary injury following SARS-CoV-2 infection, indicates that further studies incorporating more advanced pulmonary function testing and imaging modalities are warranted. Bronchodilator reversibility testing was also not performed; therefore, reversible airway obstruction could not be evaluated, particularly in participants with reduced FEV1/FVC ratios or a history of asthma. This limits the ability to distinguish fixed ventilatory impairment from potentially reversible airflow limitation.

Second, COVID-19 severity was determined retrospectively on the basis of patient- or parent-reported clinical history and available medical records. Accordingly, recall bias and misclassification of disease severity cannot be excluded, especially in participants assessed several months after the acute infection.

Additional limitations include the relatively small number of participants with asymptomatic SARS-CoV-2 infection, likely reflecting the lower detection rate in children without clinical symptoms, as well as the wide interval between infection and spirometric evaluation (1–14 months), which introduced heterogeneity into the study population. The inclusion of children with pre-existing bronchial asthma may also have influenced the interpretation of spirometric abnormalities, particularly those suggestive of airflow limitation, and the potential contribution of underlying asthma cannot be excluded.

It should also be acknowledged that, despite acceptable regression diagnostics, the relatively small number of participants with abnormal spirometry (*n* = 26) may have limited the stability and precision of the multivariable logistic regression model. Therefore, these results should be interpreted with caution and validated in larger independent cohorts.

Finally, the lack of pre-infection pulmonary function data precludes determination of whether the observed abnormalities developed after SARS-CoV-2 infection or were present beforehand. Likewise, the absence of a non-infected control group limits interpretation of whether the prevalence of spirometric abnormalities observed in this cohort differs from that expected in the general pediatric population.

## 5. Conclusions

Among the children and adolescents included in the study, pulmonary function abnormalities were identified in nearly one quarter of children and adolescents with a history of SARS-CoV-2 infection (23.85%). Reduced FVC was the most frequently observed spirometric abnormality. No significant associations were demonstrated between spirometric abnormalities and time elapsed since infection, clinical severity of COVID-19, age, or comorbidities. These findings suggest that alterations in spirometric parameters may be present in a subset of pediatric patients following SARS-CoV-2 infection; however, their clinical relevance and underlying pathophysiological mechanisms remain to be determined. In the absence of pre-infection spirometric measurements and a non-infected control group, the results should be interpreted with caution. Further longitudinal studies are warranted to better delineate the long-term respiratory sequelae of SARS-CoV-2 infection in pediatric populations and to identify factors potentially associated with persistent impairment in pulmonary function.

## Figures and Tables

**Table 1:** Frequency and types of spirometric abnormalities in the study population (*n* = 109).

Spirometry Result	*n* = Number of Patients
NORMAL, *n*	83
NORMAL, %	76.15
FEV1p, mean	56.63
FVCp, mean	43.23
FEV1/FVCp, mean	55.66
ABNORMAL, *n*	26
ABNORMAL, %	23.85
Isolated reduction in FVC, *n*	14
Isolated reduction in FVC, %	12.84
Isolated reduction in FEV1/FVC, *n*	8
Isolated reduction in FEV1/FVC, %	7.34
Concurrent reduction in FEV1 and FVC, *n*	3
Concurrent reduction in FEV1 and FVC, %	2.75
Concurrent reduction in FEV1 and FEV1/FVC, *n*	1
Concurrent reduction in FEV1 and FVC, %	0.92

**Table 2:** Multivariate analysis of factors associated with the outcome.

Predictor	OR	95% CI	*p*-Value
BMI percentile, per 10 percentile points	0.80	0.68–0.94	0.007
Age, years	1.02	0.88–1.17	0.818
Months since infection	0.98	0.82–1.17	0.837
COVID-19 severity			
Asymptomatic (reference)	1.00	-	-
Symptomatic	0.61	0.12–3.20	0.559
Mildly symptomatic	0.79	0.17–3.81	0.772

## Data Availability

The data presented in this study are available on request from the corresponding author due to ethical and privacy restrictions related to patient information.

## Abbreviations

The following abbreviations are used in this manuscript:

SARS-CoV-2	Severe Acute Respiratory Syndrome Coronavirus 2
PIMS-TS	Paediatric Inflammatory Multisystem Syndrome—Temporally associated with SARS-CoV-2
PCR	Polymerase Chain Reaction
ATS	American Thoracic Society
ERS	European Respiratory Society
GLI	Global Lung Function Initiative
FVC	Forced Vital Capacity
FEV1	Forced Expiratory Volume in 1 Second
FEV1/FVC	pseudo-Tiffeneau Index
P	value expressed in percentiles
BMI	Body Mass Index
